# PAK1 and PAK4 as therapeutic targets for Ewing sarcoma: a commentary

**DOI:** 10.46439/cancerbiology.2.032

**Published:** 2021

**Authors:** Sydney E. Parks, Jason T. Yustein

**Affiliations:** 1 Texas Children’s Cancer and Hematology Centers and The Faris D. Virani Ewing Sarcoma Center, Baylor College of Medicine, Houston, TX 77030, USA; 2 Cancer and Cell Biology Program, Baylor College of Medicine, Houston, TX 77030, USA; 3 Department of Molecular and Cellular Biology, Baylor College of Medicine, Houston, TX 77030, USA; 4 Dan L. Duncan Comprehensive Cancer Center, Baylor College of Medicine, Houston, TX 77030, USA

## Abstract

Ewing sarcoma (ES) is an aggressive pediatric bone tumor that is prone to metastasis. Due to low five-year survival rates and limited therapeutic options for metastatic disease, there is a dire clinical need for improved ES treatments. Targeting p21-activated kinases (PAKs) may be key. PAK1 and PAK4 are associated with aggressive ES and poor patient outcomes, although their molecular mechanisms remain largely uncharacterized in this disease. This commentary aims to highlight the recent advancements made to the understanding of PAK1 and PAK4 in ES in the paper “p21-activated kinases as viable therapeutic targets for the treatment of high-risk Ewing sarcoma” by Qasim et al.

## Introduction

Ewing sarcoma (ES) is the second most common pediatric bone malignancy. Unfortunately, ES is an aggressive disease that is likely to metastasize. While the current five-year survival rate for those with localized disease is approximately 65% to 75%, individuals with metastatic disease are faced with a five-year survival rate of less than 30% [1]. In an attempt to improve patient outcomes and pinpoint a more efficacious ES therapeutic target, Qasim et al. investigated the roles of p21-activated kinases (PAKs) in their paper, “p21-activated kinases as viable therapeutic targets for the treatment of high-risk Ewing sarcoma” [2].

## p21-Activated Kinases (PAKs) and Previously Identified Mechanistic Roles

PAKs make up a family of serine/threonine kinases that were first classified as effectors of Rho-family GTPases. PAKs are divided into two groups: Group I (PAK1, PAK2, and PAK3), and Group II (PAK4, PAK5, and PAK6). In healthy tissues, PAKs are broadly involved in vital processes such as cytoskeletal maintenance, cell motility, and cell survival [3]. In cancers, however, some PAKs are known to enable oncogenic and metastatic properties, such as evasion of apoptosis [4], promotion of anchorage-independent growth [4–6], inhibition of cell adhesion [5], and promotion of cell migration [7]. PAK1 and PAK4, for instance, are of particular interest in cancer biology due to their involvement in tumor formation and upregulation in various cancer types [8].

PAK4, for example, activates the Wnt signaling pathway, which is a commonly dysregulated signaling pathway in cancers. By phosphorylating β-catenin, PAK4 protects β-catenin from proteasomal degradation. This same study examined the dynamic nature of PAK4 as a shuttling protein between the cytoplasm and nucleus, and determined that the nuclear import of PAK4 was associated with greater nuclear import of β-catenin and increased downstream Wnt signaling [9]. In gastric cancer, PAK4 has also been demonstrated to promote cisplatin resistance by activating both the MAPK and PI3K/Akt pathways [10]. In rhabdomyosarcoma (RMS), analysis of transcriptomic data from RMS tumors treated with and without a PAK4 inhibitor illustrated that GTPase/Ras signaling, as well as Notch and Hedgehog signaling, were downregulated when PAK4 was inhibited [11]. PAK4, and its enzymatic activity, has also been implicated in anchorage-independent growth; kinase-dead PAK4 was shown to reduce the Ras-driven transformation of rat intestinal epithelial cells in soft agar, while wild type PAK4 rat intestinal epithelial cells experienced the expected Ras-driven transformation and anchorage-independent growth in soft agar [6]. Another study illustrated that wild type and constitutively active PAK4 constructs alone were enough to lead to NIH3T3 cell transformation and tumor formation in athymic mice [12].

PAK1 has also been implicated in pathways in cancer. Previous studies have indicated that PAK1 is necessary for cell transformation caused by Rac, Ras, and Cdc42 in the MAPK pathway [8]. Further downstream in that pathway, PAK1 phosphorylates Raf-1 and MEK1, which primes Raf-1 to subsequently phosphorylate and activate MEK1. This leads to the activation of the ERK pathway, increased oncogenic gene expression, and stimulation of cell migration [13,14]. In colon cancer, downregulation of PAK1 is associated with reduced cell proliferation and β-catenin levels. Similar to the role of PAK4 in Wnt signaling, PAK1 has been found to directly phosphorylate β-catenin, stabilizing it, and leading to increased transcriptional activity [15]. Also in colon cancer, PAK1 is a critical component of the Akt pathway, which promotes cell survival. Downregulation of PAK1 was associated with a decrease in Akt activity and reduced cell viability [14]. It has been shown that the PAK1 kinase domain serves as a scaffold that binds both Akt and PDK1, which facilitates Akt phosphorylation mediated by PDK-1. This study also indicated that PAK1 binding to Akt affects the cellular localization of Akt, which in turn alters the binding partners available to it [16].

A 2017 study by Satterfield et al. began to unravel the molecular mechanisms governing PAK1 in ES. The oncogenic properties of PAK1 were found to be activated, at least in part, by increased expression of the microRNA, miR-130b, which is associated with proliferation and increased metastatic potential in ES cells. MiR-130b activates CDC42 by directly decreasing the expression of Arghap1, which is a negative regulator of CDC42. CDC42 then binds to PAK1, leading to PAK1 autophosphorylation. Taken together, the activation of this CDC42/PAK1 signaling led to the downstream activation of the JNK pathway, as demonstrated through the upregulation of the c-FOS and c-JUN genes. JNK pathway activation causes AP-1 translocation into the nucleus, where it transcriptionally regulates various oncogenic genes [17]. The oncogenic function of CDC42 in Ewing sarcoma is further supported by a study demonstrating that CDC42 activation and upregulation is partly responsible for the role of hepatoma-derived growth factor (HDGF) in local invasion. HDGF is known to promote cell proliferation and induce the evasion of apoptosis [18].

Despite recent discoveries of the functions of PAK1 in ES, a complete understanding of the roles of PAK1 and PAK4 in ES has not yet been achieved. In an attempt to bridge this gap in knowledge, Qasim et al. conduct both *in vitro* and *in vivo* studies to shed light on the role of PAKs 1 and 4 in ES and propose possible novel ES therapeutic approaches through targeting PAKs.

## High PAK1 and PAK4 Expression Linked to Metastasis and Advanced Ewing Sarcoma

Through analysis of ES transcriptomic data from the R2: Genomics Analysis and Visualization Platform, it was found that higher PAK1 and PAK4 expression levels were associated with metastatic disease and worse patient outcome, while patients with localized disease demonstrated lower levels of PAK1 and PAK4 expression [2]. Increased PAK1 and PAK4 expression in ES is consistent with those seen in other cancer types [8]. Based on these data, PAK1 and PAK4 were then silenced *in vitro* by siRNA in ES cell lines, which led to decreased cell mobility, expansion, and cell viability, thus demonstrating the role of these PAKs in ES progression and metastasis [2].

## PAKs as Ewing Sarcoma Targets

Given the association of PAK1 and PAK4 with *in vitro* tumorigenic properties, PAK inhibitors, such as PF-3758309 (preferential PAK4 inhibitor), KPT-9274 (dual PAK4 and NAMPT inhibitor), and FRAX-597 (preferential PAK1 inhibitor), were tested in ES cell lines to determine if they would reduce the tumorigenic properties associated with PAK1 and PAK4 expression. Over a 72-hour time period, Ewing sarcoma cell lines A673, TC32, and CHLA-10 all showed high sensitivity to each of the PAK inhibitors, as demonstrated by their decreased cell viability. Each of these three drugs were synergistic when combined with treatment by chemotherapy agents such as doxorubicin, SN-38, and vincristine. Additionally, 24-hour treatment with these PAK inhibitors led to a decrease in cell mobility and invasiveness, supporting earlier findings that PAK1 and PAK4 are involved in tumorigenesis and metastasis [2].

## PAK Inhibitor Anti-Tumor Activity *In vivo*

Anterior intratibial injection of A673 and TC71 ES cells in NOD scid gamma (NSG) mice, followed by either PF-3758309 or KPT-9274 treatment, led to decreased tumor size and metastatic burden as compared to the control group of mice that received no treatment. In addition, NSG mice bearing implanted pieces of MSKEWS-66647, an ES PDX model, showed a reduced rate of tumor progression when treated with KPT-9274 over four weeks. Taken together, this *in vivo* data supports the findings of the *in vitro* studies in ES cell lines – PAK4 is involved in ES tumor-promoting processes, and its inhibition suppresses tumorigenesis [2].

## Mechanistic Signaling Effects of PAK4 Inhibition by KPT-9274 Treatment

Gene Set Enrichment Analysis (GSEA) of both control and KPT-9274-treated TC71 xenografts indicated that a variety of oncogenic pathways were suppressed in the treated tumor samples. The xenografts receiving KPT-9274 treatment showed repressed levels of genes involved in the MAPK, YAP, and Wnt signaling pathways, as well as an increase in immune stimulatory pathways, which deserve additional investigations [2].

In ES, the MAPK, YAP, and Wnt signaling pathways are associated with oncogenic properties. Both canonical and noncanonical Wnt signaling, for instance, have been shown to be key players in ES transformation and cell migration [19]. As for MAPK, ES cells exhibit increased levels of MAPK/ERK signaling and studies blocking IGF-1R, a receptor upstream of MAPK signaling, have seen reduced ES tumor growth. This demonstrates the oncogenic dependence on MAPK signaling [20]. Finally, YAP signaling is crucial for ES cell proliferation and the loss of contact inhibition. A recent study indicated that the use of a YAP/TAZ/TEAD inhibitor led to repressed cell migration in ES [21]. Taken together, the reduced expression levels of genes involved in MAPK, YAP, and Wnt signaling in xenografts following KPT-9274 treatment indicate not only that targeting PAK4 is of therapeutic significance, but that PAK4 is a hub of many oncogenic signaling pathways and its inhibition may have wide-ranging anti-tumor effects.

## Future Directions

In Qasim et al., PAKs 1 and 4 are associated with aggressive Ewing sarcoma and poor patient prognosis, and PAK4 inhibition is linked to decreased Ewing sarcoma cell viability, synergistic *in vitro* effects with chemotherapy agents, and decreased tumorigenic and metastatic potential *in vivo*. Given the role of PAKs in the oncogenic and metastatic properties of Ewing sarcoma and the clinical need for improved therapeutics for metastatic disease, it is crucial to build upon our current understanding of the functions of PAKs in Ewing sarcoma [2].

While Qasim et al. provide important insights into the overarching effects of PAK1 and PAK4 expression in Ewing sarcoma, there is still much to be discovered from a mechanistic standpoint. For instance, it is known that PAK4 travels between the cytoplasm and nucleus [9]. However, it is not yet known if and how the various functions of PAK4 differ between those two locations, and if this makes a difference to ES progression. Additionally, given the importance of PAK4 in metastatic disease, studies should be conducted to understand if the role of PAK4 differs in an ES cell of a primary tumor *vs*. an ES cell at a metastatic site. Understanding the specific roles of PAK4 in metastatic ES signaling would highlight potentially novel therapeutic targets for advanced and aggressive disease.

Finally, future studies could include an investigation into the role of PAK4 and immune evasion in Ewing sarcoma. Recent studies have noted a potential critical role for PAK4 in directly regulating immune checkpoint expression as highlighted in a review by Naija et al. [22]. One prior study indicated that PAK4 expression levels are negatively correlated to immune cell infiltration in numerous human cancer types. In melanoma, tumors with greater PAK4 expression were less responsive to anti-PD-1 therapy. Additionally, it was shown that anti-tumor immune cell infiltration was increased following PAK4 knockdown, whereas wild type tumors remained devoid of immune cell infiltration [23]. PAK4 regulation of Wnt/β-catenin is one potential mechanism for alterations in the immune infiltration, but further defining the molecular mechanisms of PAK4 and immune modulation could significantly aid in designing effective immunotherapies for ES tumors and other malignancies with high PAK4 expression.
